# Phase I study of primary treatment with 5-FU, oxaliplatin, irinotecan, levofolinate, and panitumumab combination chemotherapy in patients with advanced/recurrent colorectal cancer involving the wild-type *RAS* gene: the JACCRO CC-14 study

**DOI:** 10.1007/s10147-017-1228-5

**Published:** 2018-02-20

**Authors:** Hironaga Satake, Akihito Tsuji, Masato Nakamura, Masaaki Ogawa, Takeshi Kotake, Yukimasa Hatachi, Hisateru Yasui, Akinori Takagane, Yoshihiro Okita, Kumi Nakamura, Toshihide Onikubo, Masahiro Takeuchi, Masashi Fujii

**Affiliations:** 1 Department of Medical Oncology, Kobe City Medical Center General Hospital, Kobe, Japan; 2 Department of Clinical Oncology, Kagawa University Faculty of Medicine–Kagawa University Hospital, 1750-1, Ikenobe, Miki-cho, Kita-gun, Kagawa 761-0793 Japan; 3 Aizawa Comprehensive Cancer Center, Aizawa Hospital, Matsumoto, Japan; 4 Department of Surgery, Kazuno Kosei Hospital, Kazuno, Japan; 5 Department of Surgery, Hakodate Goryoukaku Hospital, Hakodate, Japan; 6 Department of Clinical Medicine (Biostatistics), Kitasato University School of Pharmacy, Tokyo, Japan; 7 Japan Clinical Cancer Research Organization, Tokyo, Japan

**Keywords:** FOLFOXIRI, Panitumumab, Triplet chemotherapy, Metastatic colorectal cancer, *RAS*

## Abstract

**Background:**

FOLFOXIRI is now regarded as the chemotherapy regimen that offers the best platform for the treatment of colorectal cancer. However, the safety and efficacy of FOLFOXIRI + panitumumab has not been demonstrated. We conducted a phase I study to determine the recommended dose of FOLFOXIRI + panitumumab as first-line treatment for *RAS* wild-type metastatic colorectal cancer (mCRC).

**Methods:**

Patients received combination therapy consisting of panitumumab (6 mg/kg on day 1) + FOLFOXIRI [irinotecan (CPT-11), oxaliplatin (L-OHP) 85 mg/m^2^, and folinate (LV) 200 mg/m^2^] on day 1, followed by fluorouracil (5-FU) 3200 mg/m^2^ infused as a 46-h continuous infusion starting on day 1) repeated every 2 weeks as first-line treatment of *RAS* wild-type mCRC patients. A decrease in CPT-11 dose was planned (started at level 1: CPT-11 165 mg/m^2^).

**Results:**

Seven patients were enrolled, and six were assessed for safety and efficacy. Maximum tolerated dose was not reached at level 1; all patients were treated at these levels. The common Grade 3 or 4 relevant toxicities were diarrhea (50%), hypokalemia (33%) and stomatitis (33%). No treatment-related deaths occurred. Of the six patients assessed four had partial response and the two others had stable disease; hence, the response rate was 66.7% (95% confidence interval 28.9–100%) and the disease control rate was 100%. Time to protocol treatment failure was 7.2 (1.4–7.3) months.

**Conclusion:**

The FOLFOXIRI + panitumumab chemotherapy regimen was well tolerated by our patients with mCRC and showed promising anti-tumor activity. The recommended phase II dose was determined to be the same as the standard doses of this regimen used worldwide.

## Introduction

During the last decade the annual rate of colorectal cancer (CRC) in Japan has been steadily increasing. According to one 2014 statistical prediction of cancer incidence, the number of morbidities from CRC (colonic cancer plus rectal cancer) will ultimately reach approximately 130,000 [1]. In developed Western countries, CRC ranks as the second-leading cause of death, following lung cancer. Globally, the development of methods for the prevention, early diagnosis, and treatment of CRC remains a matter of urgent concern.

Chemotherapy is the first-line treatment for unresectable metastatic CRC (mCRC), and the greater availability of the key drugs fluorouracil (5-FU), irinotecan (CPT-11), and oxaliplatin (L-OHP) has been shown to be positively correlated with the greater prolongation of prognosis [2]. The efficacy of the FOLFOXIRI [5-FU, leucovorin (LV), L-OHP, CPT-11] and FOLFIRI (5-FU, LV, CPT-11) chemotherapy regimens has been compared, with the FOLFOXIRI regimen proving to be significantly better in terms of response rate (RR), progression-free survival (PFS), and overall survival (OS) [3]. In addition, the results of the TRIBE study established the FOLFOXIRI + bevacizumab combination regimen as standard therapy for patients with mCRC [4]. Accordingly, FOLFOXIRI ± bevacizumab therapy is now regarded as a first-line treatment for mCRC. However, there are several aspects of the FOLFOXIRI regimen which require attention. One of these is the CPT-11 dosage in FOLFIRI therapy in Japan, which at 150 mg/m^2^ is lower than the 180 mg/m^2^ used in Western countries. On the other hand, despite L-OHP being used concomitantly in FOLFOXIRI therapy, the dose of irinotecan in the FOLFOXIRI regimen is set at 165 mg/m^2^ in Japan, the same as that in Western countries and higher than that for FOLFIRI therapy in Japan. We previously reported that the combination therapy regimen of FOLFOXIRI + bevacizumab could be administered in the same way as global standard doses [L-OHP 85 mg/m^2^; CPT-11 165 mg/m^2^, LV 200 mg/m^2^, 5-FU 3,200 mg/m^2^, infused as a 46-h continuous infusion + bevacizumab 5 mg/kg] in Japanese patients [5]. This was confirmed in the QUATTRO trial (No. UMIN000013797), a phase II study which evaluated a global standard regimen of FOLFOXIRI + bevacizumab as first-line therapy in patients with mCRC. The results of this trial showed that the standard dose of FOLFOXIRI could be administered safely. Furthermore, the combination of panitumumab + FOLFOX therapy or FOLFIRI therapy is recommended as the primary treatment for patients with mCRC with the wild-type* RAS* gene. Although the combination of panitumumab + FOLFOXIRI therapy achieves a high response rate, the addition of panitumumab to the FOLFOXIRI regimen has been shown to increase the incidence and severity of diarrhea [6]. Hence, the dosage and safety of FOLFOXIRI + panitumumab have not yet been established.

In the study reported here, we conducted a phase I study to determine the recommended dose (RD) of CPT-11 in FOLFOXIRI therapy.

## Patients and methods

### Eligibility criteria

Eligibility criteria were age ≥ 18 years at the time of registration; histologically identified CRC (adenocarcinoma); unresectable advanced/recurrent CRC involving the wild-type *KRAS* or *RAS* gene; evaluable lesion confirmed by objective methods, such as computed tomography (CT) within 30 days prior to registration; no prior chemotherapy for unresectable primary tumor and distant metastases or lymph node metastases; Eastern Cooperative Oncology Group (ECOG) performance status 0 or 1; adequate organ function, as defined by a white cell blood (WBC) count of 3000–12,000/mm^3^, absolute neutrophil count of ≥ 2000/mm^3^, hemoglobin ≥ 10 g/dl, platelet count of ≥ 10.0 × 10^4^/mm^3^, total bilirubin ≤ 1.5 mg/dl, serum transaminase levels of ≤ 100 U/l (≤ 200 IU/l for patients with hepatic metastases), and serum creatinine level of ≤ 1.5 mg/dl. Exclusion criteria were evidence of prior chemotherapy with panitumumab, oxaliplatin, or irinotecan; prior myocardial infarction within 3 months; history of unstable angina pectoris, interstitial pneumonia, fibroid lung, or severe emphysema; concurrent active malignancy; uncontrolled infection; severe mental disorder; peripheral sensory neuropathy > Grade 2; pregnancy or lactation.

This trial was carried out in accordance with the Helsinki Declaration and Ethical Guidelines for Clinical Studies and was approved by the institutional review boards of all participating institutions. All patients were required to give written informed consent before entering the study. Japan Clinical Cancer Research Organization (JACCRO) conducted the data management, central monitoring, and statistical analysis.

### Study design and treatment

The protocol treatment was defined as chemotherapy consisting of L-OHP, CPT-11, LV, 5-FU, plus panitumumab. Specifically, the treatment regimen consisted of a 1-h intravenous (i.v.) administration of panitumumab, 1-h administration of CPT-11, 2-h concomitant infusion of L-OHP 85 mg/m^2^ and LV 200 mg/m^2^, followed by 46-h continuous i.v. administration of 5-FU 3200 mg/m^2^. This treatment regimen was repeated for a maximum of 12 courses, with 2 weeks regarded as one course. The study was designed to evaluate the maximum tolerated dose (MTD) of combination therapy with 5-FU, L-OHP, CPT-11, LV, and panitumumab as an initial treatment in patients with unresectable mCRC involving the wild-type *KRAS* or *RAS* gene, and to determine the RD. Six patients were treated at dose level 1 (L-OHP 85 mg/m^2^, CPT-11 165 mg/m^2^, LV 200 mg/m^2^, 5-FU 3,200 mg/m^2^, infused as a 46-h continuous infusion, and panitumumab 6 mg/kg). If three or more of the six patients experienced a dose limiting toxicity (DLT), six additional patients were accrued at the next lower dose level (L-OHP 85 mg/m^2^, CPT-11 150 mg/m^2^, LV 200 mg/m^2^, 5-FU 3,200 mg/m^2^, infused as a 46-h continuous infusion, and panitumumab 6 mg/kg). Among the levels at which a DLT was observed in more than three of the six patients, the lowest level was estimated to be the MTD. If an MTD was not observed in three or more of the six patients at dose level 1, level 1 or higher was estimated to be the MTD. One level below the MTD was tentatively determined to be the RD. If the MTD was estimated to be level 1 or higher, level 1 was tentatively determined to be the RD.

The DLT was defined as any one or more of the following adverse events occurring during the DLT evaluation period: (1) neutropenia of Grade 4 (neutrophil count < 500/mm^3^) persisting for ≥ 4 days; (2) thrombocytopenia of Grade 4 (platelet count < 2.5 × 10^4^/mm^3^); (3) febrile neutropenia with a fever of ≥ 38.5 °C; (4) clinically significant non-hematotoxicity of Grade 3 or higher; (5) discontinuation of treatment due to an adverse event; (6) any death for which a possible association with treatment could not be ruled out. The DLT evaluation period was the period from drug administration on Day 1 of the first course to before drug administration on Day 1 of the second course. Treatment was administered biweekly until evidence of progression, unacceptable toxicity, or patient refusal. Treatment was delayed if, on the planned day of treatment, the WBC was < 2000/mm^3^, neutrophil level was < 1000/mm^3^, platelet count was < 7.5 × 10^4^/mm^3^, hemoglobin level was < 9.0 g/dl, serum bilirubin level was > 1.5 mg/dl, or persistent diarrhea or stomatitis, skin symptoms, or peripheral sensory or motor neurotoxicity higher than Grade 1 (NCI-CTC) was present. In the event of peripheral sensory or motor neurotoxicity ≥ Grade 3, treatment with L-OHP was interrupted. In the event of Grade 4 non-hematologic toxicities, treatment was definitively interrupted.

To prevent chemotherapy-induced nausea and vomiting (CINV), the 5-hydroxytryptamine 3 (5HT_3_) antagonist dexamethasone was administered at 9.9 mg i.v., and neurokinin 1 receptor antagonists (aprepitant, fosaprepitant) were administered before chemotherapy. Prophylactic use of granulocyte colony-stimulating factor (G-CSF) was not allowed. No after-treatment was specified in cases of completion or discontinuation of the protocol treatment.

### Study assessment

Pretreatment evaluation included a medical history; physical examination; complete blood cell count and serum chemistry tests; colonoscopy; chest, abdominal and pelvic CT scans. Clinical examination and biochemical tests were required before and during each cycle. All adverse events experienced during the study were recorded and graded according to the National Cancer Institute Common Terminology Criteria for Adverse Events (CTCAE version 4.0; https://ctep.cancer.gov/). Response, as determined by the investigators, was recorded according to Response Evaluation Criteria In Solid Tumors (RECIST) criteria v1.1 (http://www.irrecist.com/).

### Endpoints

The primary endpoint in this study was the MTD and RD of this regimen. Secondary endpoints included RR, time to treatment failure (TTF), PFS, OS, treatment completion rate (12 courses), and safety. Dose intensity was calculated as the ratio of the actual to planned dose intensity in milligrams per square meter per week. The survival curve was estimated using the Kaplan–Meier method. Safety and efficacy analyses were both conducted on an intention-to-treat population, defined as all patients enrolled in the study who received at least one dose of chemotherapy. TTF was defined as the time/date at which any one of the following occurred the earliest: date at which aggravation was diagnosed, date of death due to any cause, or date of completion or discontinuation of protocol treatment, using the day of registration as the base date. PFS was defined as the time to the earlier of the date at which the aggravation was diagnosed or the date of death due to any cause, with the day of registration as the base date. OS was determined as the time to date of death due to any cause or last confirmation of survival, with the day of registration as the base date. Statistical data were obtained using the SAS software, version 9.2 (SAS Institute Inc., Cary, NC).

This trial was registered with the University Hospital Medical Information Network (No. UMIN000015475).

## Results

### Patients

**Fig. 1:**
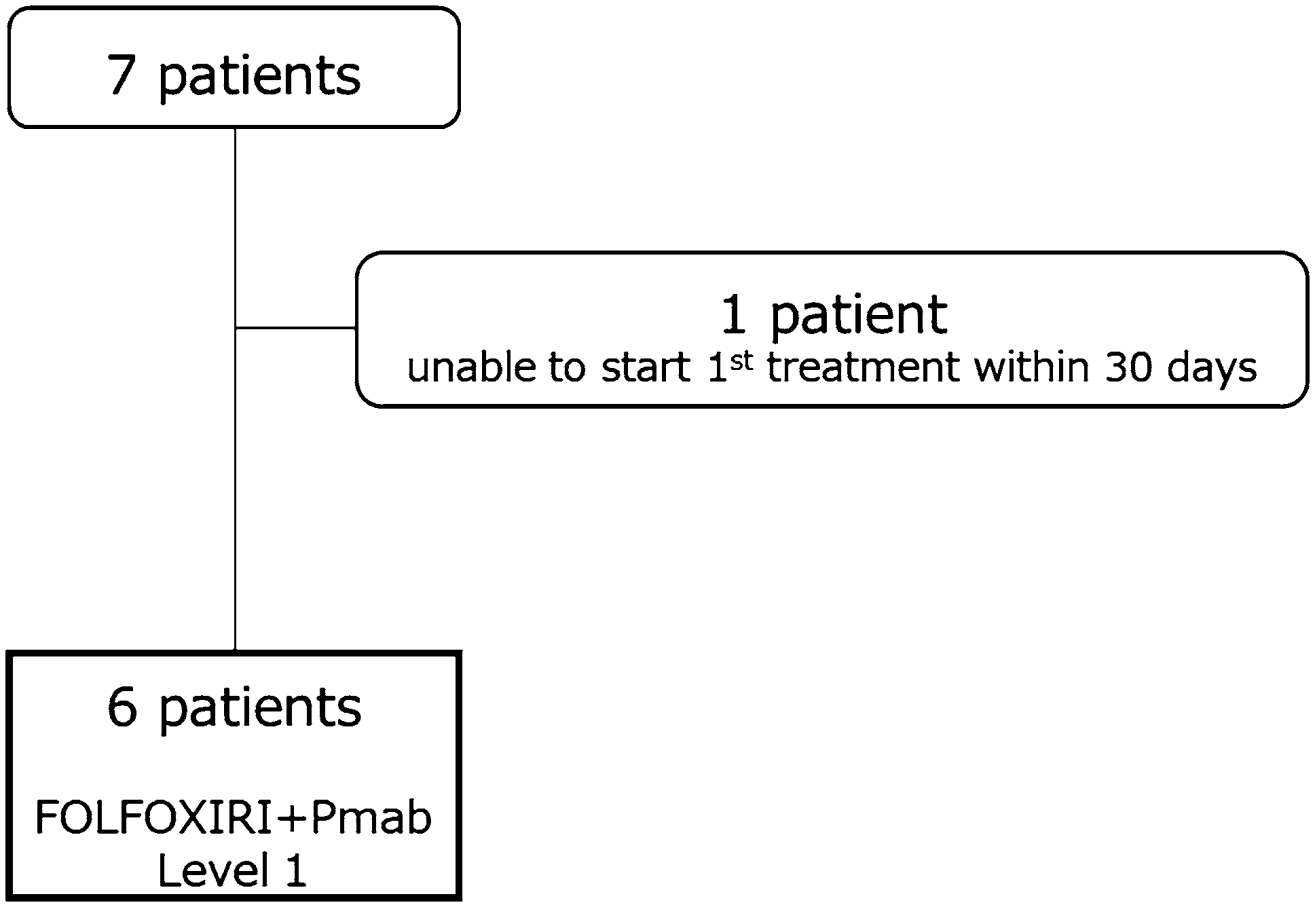
Flow diagram of patient enrollment. *Pmab* Panitumumab

From January 2015 to July 2015, a total of seven patients were enrolled. One patient was excluded from further treatment and all analyses because the patient could not start protocol treatment within 30 days of enrollment, leaving six patients for assessment (Fig. 1). Characteristics of the patients are listed in Table 1. All patients were chemo-naïve and had a good performance status. All six patients had histologically proven adenocarcinoma, of whom two had the *KRAS* exon2 wild-type and four had the *KRAS*, *NRAS, HRAS* wild-type. The primary tumor locations were left-sided (distal to splenic flexure) in four patients and right-sided (proximal to splenic flexure) in two patients.

**Table 1:** Patient characteristics (*n* = 6)

Variables	Values
Age (years)	60 (38–74)
Sex	
Male	3 (50)
Female	3 (50)
ECOG PS	
0	6 (100)
1	0 (0)
RAS status	
* KRAS* wt	2 (33)
* RAS* wt	4 (67)
UGT1A1	
WT	4 (67)
Single hetero	2 (33)
Cancer status	
Advanced	4 (67)
Relapse	2 (33)
Primary tumor	
Colon	4 (67)
Rectum	1 (17)
Other	1 (17)
TNM T stage^a^	
T3	2 (33)
T4a	3 (50)
T4b	1 (17)
TNM N stage^a^	
N0	1 (17)
N1a	1 (17)
N1b	2 (33)
N2b	2 (33)
TNM M stage^a^	
M1a	1 (17)
M1b	5 (83)

Values in table are presented as a number with the percentage in parenthesis, with the exception of Age which is presented as the median with the range in parenthesis

*ECOG* Eastern Cooperative Oncology Group, *PS* performance status, *wt* wild type, *WT* wild type

^a^TNM classification according to the Union for International Cancer Control (UICC) TNM classification of malignant tumors 7th edition (Wiley, Hoboken)

### Treatment administration, DLT, RD and dose intensity

Six patients were treated at dose level 1 (L-OHP 85 mg/m^2^, CPT-11 165 mg/m^2^, l-LV 200 mg/m^2^, 5-FU 3,200 mg/m^2^, infused as a 46-h continuous infusion, and panitumumab 6 mg/kg). No patient had a DLT at level 1, and the RD was determined to be L-OHP 85 mg/m^2^, CPT-11 165 mg/m^2^, LV 200 mg/m^2^, and 5-FU 3,200 mg/m^2^ infused as a 46-h continuous infusion + panitumumab 6 mg/kg.

Four patients completed the protocol treatment of 12 cycles (67%). Two patients discontinued the protocol treatment, one due to disease progression and one due to adverse events. All patients required a dose reduction for CPT-11 at a median of the fourth cycle, mainly due to diarrhea. Four patients needed a reduction of the L-OHP dose at a median of the third cycle due to neuropathy. All patients needed a reduction of the 5-FU dose at a median of the fourth cycle, mainly due to diarrhea or stomatitis. Two patients needed a reduction of the panitumumab dose at a median of the sixth cycle due to paronychia and stomatitis. The relative dose intensity delivered was 81.1% for L-OHP, 88.1% for CPT-11, 97.2% for l-LV, 90.4% for 5-FU, and 83.7% for panitumumab, with a median of 12 cycles per patient (range 3–12 cycles).

### Toxicity

The worst toxicity during the DLT evaluation period and throughout the treatment period is listed in Tables 2 and 3, respectively. All six patients who received protocol treatment were assessed for safety. Non-hematological adverse events of Grade ≥ 3 occurred in none of patients during the DLT evaluation period, and Grade 3 or higher neutropenia and febrile neutropenia did not occur in any of the patients through the treatment period. Common Grade 3 or higher relevant toxicities were diarrhea (50%), hypokalemia (33%), and stomatitis (33%). No treatment-related death was observed.

**Table 2:** Maximum toxicity per patient during the dose limiting toxicity evaluation period (*n* = 6)

Adverse event	NCI-CTC grade	
	All grades, *n* (%)	≥ Grade 3, *n* (%)
Hematologic		
Leukopenia	0 (0)	0 (0)
Neutropenia	0 (0)	0 (0)
Thrombocytopenia	0 (0)	0 (0)
AST increased	1 (17)	0 (0)
ALT increased	1 (17)	1 (17)
Hypoalbuminemia	2 (33)	0 (0)
Hypokalemia	2 (33)	0 (0)
Hyponatremia	1 (17)	1 (17)
Hypomagnesemia	2 (33)	0 (0)
Non-hematologic		
Alopecia	2 (33)	–
Anorexia	3 (50)	0 (0)
Conjunctivitis	1 (17)	0 (0)
Constipation	1 (17)	0 (0)
Diarrhea	4 (67)	0 (0)
Dry skin	2 (33)	0 (0)
Fatigue	1 (17)	0 (0)
Febrile neutropenia	0 (0)	0 (0)
Infusion-related reaction	1 (17)	0 (0)
Malaise	3 (50)	–
Nausea	3 (50)	0 (0)
Palpitations	1 (17)	0 (0)
Peripheral sensory neuropathy	3 (50)	0 (0)
Pruritus	1 (17)	0 (0)
Rash acneiform	3 (50)	0 (0)
Stomatitis	2 (33)	0 (0)
Vomiting	1 (17)	0 (0)
Weight loss	1 (17)	0 (0)

*NCI-CTC* National Cancer Institute Common Toxicity Criteria, *AST* aspartate transaminase,* ALT*, alanine transaminase

**Table 3:** Maximum toxicity per patient during the overall treatment period (*n* = 6)

Adverse event	NCI-CTC grade	
	All grades, *n* (%)	≥ Grade 3, *n* (%)
Hematologic		
Leukopenia	5 (83)	0 (0)
Neutropenia	5 (83)	0 (0)
Thrombocytopenia	1 (17)	0 (0)
Anemia	2 (33)	0 (0)
AST increased	1 (17)	0 (0)
ALT increased	3 (50)	1 (17)
Alkaline phosphatase increased	2 (33)	0 (0)
Hyperkalemia	1 (17)	0 (0)
Hypermagnesemia	1 (17)	1 (17)
Hypoalbuminemia	3 (50)	0 (0)
Hypocalcemia	2 (33)	1 (17)
Hypokalemia	4 (67)	2 (33)
Hyponatremia	1 (17)	1 (17)
Hypomagnesemia	6 (100)	1 (17)
Proteinuria	1 (17)	0 (0)
Non-hematologic		
Abdominal pain	1 (17)	0 (0)
Alopecia	4 (67)	–
Anorexia	4 (67)	0 (0)
Anal hemorrhage	1 (17)	0 (0)
Cheilitis	1 (17)	0 (0)
Conjunctivitis	2 (33)	0 (0)
Constipation	3 (50)	0 (0)
Diarrhea	6 (100)	3 (50)
Dry skin	5 (83)	0 (0)
Dysgeusia	5 (83)	–
Fatigue	2 (33)	0 (0)
Febrile neutropenia	0 (0)	0 (0)
Fever	1 (17)	0 (0)
Hypertension	1 (17)	0 (0)
Infusion-related reaction	3 (50)	0 (0)
Malaise	5 (83)	–
Nausea	5 (83)	0 (0)
Palpitations	1 (17)	0 (0)
Paronychia	3 (50)	0 (0)
Peripheral sensory neuropathy	6 (100)	0 (0)
Pruritus	1 (17)	0 (0)
Rash acneiform	5 (83)	0 (0)
Skin hyperpigmentation	2 (33)	–
Stomatitis	5 (83)	2 (33)
Urticaria	1 (17)	0 (0)
Vomiting	2 (33)	0 (0)
Weight loss	2 (33)	0 (0)

### Efficacy and survival

**Fig. 2:**
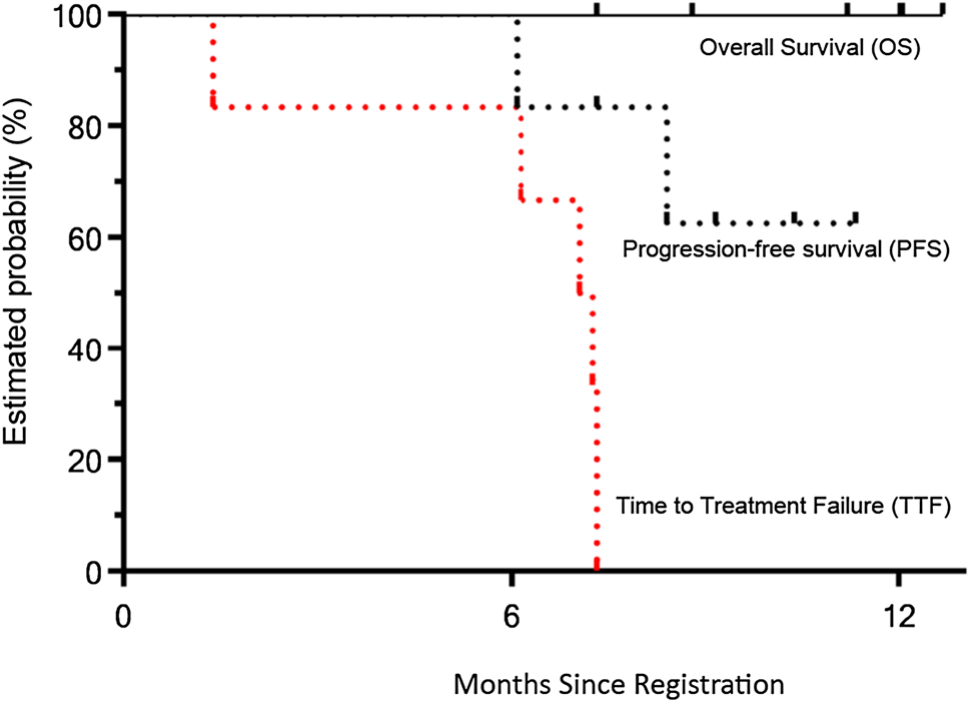
Time to treatment failure, progression-free survival, and overall survival

Response was assessable in all six patients who received the protocol treatment. No complete response was observed, and partial response was observed in four patients, giving a RR of 66.7% [95% confidence interval (CI) 28.9–100%]. The other two patients had stable disease. There was no patient with progressive disease as best response. At the time of analysis, all six patients were alive. With a median follow-up period of 11.6 months, median TTF was 7.2 months (95% CI 1.4–7.3), and neither median PFS nor OS was reached (Fig. 2).

## Discussion

In this study, we determined that the RD of FOLFIXIRI + panitumumab in patients with mCRC was L-OHP 85 mg/m^2^, CPT-11 165 mg/m^2^, LV 200 mg/m^2^, 5-FU 3,200 mg/m^2^, all infused as a 46-h continuous infusion, and panitumumab 6 mg/kg. These are the same as the standard doses for this regimen used worldwide. To our knowledge, this is the first report of the feasibility and activity of FOLFIXIRI + panitumumab in Asian patients with mCRC.

A FOLFOXIRI + anti-epidermal growth factor receptor (EGFR) antibody chemotherapy regimen for mCRC has been investigated in several prospective trials and shown to have promising efficacy, with a high RR and prolonged survival. Nevertheless, the recommended doses have not yet been determined. In a phase II trial evaluating the FOLFOXIRI + cetuximab regimen on 13 wild-type* KRAS* exon 2 codon 12 mCRC patients, the RR, median PFS, and median OS were 70% and 10.2 and 30.3 months, respectively, although there were high rates of Grade ≥ 3 neutropenia (23%), diarrhea (53%), and stomatitis (10%) [7]. In another phase II trial evaluating FOLFOXIRI + panitumumab for *RAS*, *BRAF* wild-type mCRC patients demonstrated a particularly high RR of 89% and median PFS of 11.3 months, with increased incidence and severity of neutropenia (≥ Grade 3, 48%), diarrhea (≥ Grade 3, 35%), and stomatitis (≥ Grade 3, 14%) [6]. Due to the increasing incidence of severe hematological and non-hematological toxicities, the dosage of FOLFOXIRI + anti-EGFR antibody regimens ranged from CPT-11 150–180 mg/m^2^, L-OHP 65–85 mg/m^2^, and 5-FU 1200–3200 mg/m^2^ with/without bolus infusion [6–8]. In the present study, half of the patients experienced ≥ Grade 3 diarrhea. However, scopolamine in combination with anticholinergic medicine would likely be effective from the following cycle onwards; on this basis, the recommended phase II dose for FOLFOXIRI + panitumumab was determined to be the same as the standard doses for this regimen used worldwide.

Our results show a smaller proportion of Grade 3 or more hematologic toxicities than has been reported previously. Our previous phase I study of FOLFOXIRI + bevacizumab identified the same recommended dose of FOLFOXIRI as did this study, but in the former we reported an incidence of ≥ Grade 3 neutropenia of 67% [5]. The authors of another phase I study of FOLFOXIRI for Japanese patients concluded that a modified dosage might be feasible [9]. Patients with homozygosity for UGT1A1*28 (*28/*28) or UGT1A1*6 (*6/*6) and heterozygosity for both UGT1A1*28 and *6 (*28/*6), which are associated with severe CPT-11-related neutropenia in Japanese patients, were coincidentally not enrolled in this study. The PRIME study, a phase III trial of FOLFOX with or without panitumumab for mCRC, showed that ≥ Grade 3 neutropenia did not increase when panitumumab was added to chemotherapy regimen [10]. To the contrary, a meta-analysis indicated that bevacizuamb is associated with an increased risk of neutropenic events [11]. These results mean that panitumumab added to chemotherapy does not increase the risk of neutropenia, but that bevacizumab does increase the risk of neutropenia. Furthermore, all patients needed a reduction in both CPT-11 and 5-FU dose due to gastrointestinal toxicities (diarrhea or stomatitis). This might explain why no patients in our study experienced ≥ Grade 3 neutropenia. FOLFOXIRI should be started at the recommended dose with prompt dose modifications as needed for adverse events. This modification suggests that these recommended doses are feasible.

A limitation of our present study related to study design should be discussed. We planned a de-escalation design for this study, but the dose of CPT-11 of FOLFOXIRI did not reach the MTD. The question therefore remains whether the dosage of CPT-11 could have been escalated higher in combination with panitumumab.

The association of an anti-EGFR antibody in combination with FOLFOXIRI would be a promising treatment in *RAS* wild-type mCRC patients and deserves further validation in prospective trials. In the present study, the recommended phase II dose was determined to be L-OHP 85 mg/m^2^, CPT-11 165 mg/m^2^, LV 200 mg/m^2^, 5-FU 3,200 mg/m^2^, infused as a 46-h continuous infusion, and panitumumab 6 mg/kg. The randomized phase II JACCRO CC-13 study, titled the DEEPER trial, is now recruiting patients and is evaluating the addition of bevacizumab or cetuximab to FOLFOXIRI as a first-line therapy in Japanese patients with *RAS* wild-type mCRC (UMIN000018217).
